# Selected adverse events during the 12-week induction period of a phase III trial of vunakizumab: an exploratory *post-hoc* analysis

**DOI:** 10.3389/fimmu.2026.1894231

**Published:** 2026-10-14

**Authors:** Zhendong Wei, Lu Xu, Aoxue Wang

**Affiliations:** Department of Dermatology, The Second Hospital of Dalian Medical University, Dalian, China

**Keywords:** adverse events, moderate-to-severe plaque psoriasis, risk factors, skin and subcutaneous tissue diseases, vunakizumab

## Abstract

**Objective:**

This study aimed to investigate the impact of vunakizumab on adverse events (AEs) in patients with moderate-to-severe plaque psoriasis.

**Methods:**

This exploratory *post-hoc* analysis used data from a phase III trial (NCT04839016). A total of 690 patients were included. The primary outcome was any AE occurrence during the 12-week induction treatment period.

**Results:**

The incidence of any AE during the induction period was 69.9%. No significant difference in the incidence of any AE was observed between the two groups [risk difference (RD): -2.6%; 95% confidence interval (CI): -9.8% to 4.6%]. Sensitivity analyses showed that vunakizumab was not an independent influencing factor for the occurrence of any AE [odds ratio (OR): 0.828; 95% CI: 0.574 to 1.193; *P* = 0.311]. At the system organ class (SOC) level, investigations, metabolic and nutritional disorders, infectious and parasitic diseases, and skin and subcutaneous tissue diseases had incidences of 31.9%, 26.7%, 18.0%, and 14.6%, respectively. At the preferred term (PT) level, incidences were 11.3% for hyperuricemia, 7.1% for elevated liver enzymes, and 5.1% for injection site reaction. At the SOC level, the incidence of skin and subcutaneous tissue diseases was lower in the vunakizumab group than in the placebo group (RD: -6.2%; 95% CI: -12.1% to -0.3%). At the PT level, the incidence of injection site reaction was higher in the vunakizumab group than in the placebo group (RD: 4.3%; 95% CI: 1.4% to 7.3%). Notably, after excluding psoriasis exacerbation, the association between vunakizumab and skin and subcutaneous tissue diseases was no longer significant.

**Conclusion:**

Vunakizumab is not significantly associated with the occurrence of any AE or most selected AE categories in patients with moderate-to-severe plaque psoriasis, whereas vunakizumab is associated with a higher likelihood of injection site reaction and a lower likelihood of skin and subcutaneous tissue diseases. The observed lower incidence of skin and subcutaneous tissue diseases may largely reflect improved control of psoriasis rather than a specific safety benefit of vunakizumab.

**Clinical trial registration:**

https://clinicaltrials.gov/, identifier NCT04839016.

## Introduction

Plaque psoriasis, the most common subtype of psoriasis, is characterized by red, raised areas of skin, often covered with silvery-white scale (1). Biologics have greatly changed the treatment paradigm for plaque psoriasis (2–4). Due to the favorable efficacy, the Chinese guidelines and guidelines from the American Academy of Dermatology-National Psoriasis Foundation have recommended biologics as a fundamental treatment for patients with plaque psoriasis (5, 6). However, the use of biologics raises safety concerns due to the frequent occurrence of some adverse events (AEs), such as nasopharyngitis, injection site reactions, and upper respiratory tract infections, which may further impair patients’ quality of life and affect treatment outcomes (7, 8). As a result, a deep understanding of the safety of biologics is crucial to improving the management of patients with plaque psoriasis.

Vunakizumab, a novel interleukin (IL)-17A inhibitor, has been approved in China for the treatment of moderate-to-severe plaque psoriasis (9). A phase II trial reported that the 12-week efficacy of vunakizumab was good in patients with moderate-to-severe plaque psoriasis (10). A recently completed phase III trial (NCT04839016) further demonstrated that the favorable efficacy of vunakizumab could be maintained until week 52 in patients with moderate-to-severe plaque psoriasis (11). In the previous phase III trial, we noted that some AEs (such as hyperlipidemia and hypertriglyceridemia) seemed more frequent during the induction period after vunakizumab than placebo (11).

This exploratory *post-hoc* analysis aimed to further investigate the impact of vunakizumab on the occurrence of AEs in patients with moderate-to-severe plaque psoriasis.

## Materials and methods

### Research design

This was an exploratory *post-hoc* analysis based on a previously randomized, double-blind, placebo-controlled phase III clinical trial (NCT04839016) evaluating the efficacy and safety of vunakizumab in patients with moderate-to-severe chronic plaque psoriasis. During the 12-week induction period, enrolled patients were randomly assigned to receive subcutaneous 240 mg vunakizumab or matching placebo at weeks 0, 2, 4, and 8. At week 12, patients initially randomized to the placebo group switched to vunakizumab 240 mg at weeks 12, 14, and 16 and every 4 weeks thereafter, whereas patients initially assigned to the vunakizumab group entered the maintenance period with vunakizumab administered every 4 weeks, with an additional placebo dose at week 14 to maintain blinding. All patients in the phase III study were included in this exploratory *post-hoc* analysis.

The original phase III study gained approval from the Ethics Committee. Detailed information on the participating centers and the corresponding ethics approvals for the original phase III study is provided in **Supplementary Table 1**. This exploratory *post-hoc* analysis used anonymized data from the original trial and involved no additional participant intervention, treatment assignment, or collection of new personal data. No separate waiver of ethical review or expedited ethical approval was obtained specifically for this exploratory *post-hoc* analysis. All patients signed written informed consent. The informed consent form stated that participants’ personal data, particularly medical results, would be collected, stored, and evaluated. Therefore, the use of anonymized trial data for this exploratory *post-hoc* analysis was covered by the original informed consent, and no separate informed consent was obtained specifically for this exploratory *post-hoc* analysis.

### Data preparation

This exploratory *post-hoc* analysis was performed using anonymized data from the original phase III study. Treatment (vunakizumab vs. placebo) was screened. Additionally, demographics and information related to psoriasis were screened, which included age, sex, body mass index (BMI), smoking, family history of psoriasis, previous local treatment, previous non-biological agent treatment, previous biological agent treatment, previous phototherapy, hypertension, diabetes mellitus (DM), duration of psoriasis, psoriasis area and severity index (PASI) score, static Physician’s Global Assessment (sPGA) score, body surface area (BSA), dermatology life quality index (DLQI) score.

### Outcomes

The primary outcome was any AE occurrence during the 12-week induction treatment period (as defined in the original phase III study protocol) (11). Patients were considered to have experienced any AE if at least one AE preferred term (PT) was reported during the induction treatment period. Secondary outcomes were selected AE categories at the system organ class (SOC) level and PT level. At the SOC level, the selected AE categories included metabolic and nutritional disorders, investigations, infectious and parasitic diseases, and skin and subcutaneous tissue diseases. At the PT level, the selected AEs included elevated liver enzymes, hyperuricemia, and injection site reaction. These AE categories were selected based on their clinical relevance and clinical considerations of particular concern to clinicians. For each SOC-level outcome, patients were considered to have experienced the event if at least one PT classified within the corresponding SOC was reported during the induction treatment period. The specific PTs included in each SOC-level category are provided in **Supplementary Table 2**. Coding of all AEs was performed with Medical Dictionary for Regulatory Activities (MedDRA) version 25.0.

### Statistics

Data were summarized descriptively: continuous data as mean ± standard deviation (SD) and categorical data as frequency (%). Besides, between-group comparisons employed t-test, Mann-Whitney U test, or *χ^2^* test, as applicable. Between-group differences in the incidence of AEs were expressed as risk differences (RDs), with corresponding 95% confidence intervals (CIs). Because treatment assignment was randomized in the original phase III trial, unadjusted between-group comparisons were considered the primary analyses. Analyses of individual AE categories were conducted *post hoc* and were considered exploratory. To identify factors associated with outcomes, univariable and enter-method multivariable logistic regression analyses were additionally conducted as sensitivity analyses. The univariable analysis first assessed each variable’s crude association with AE. Then, all examined variables were simultaneously entered into the multivariable regression to evaluate independent influencing factors after mutual adjustment. A multicollinearity test was conducted among variables included in the model. A variance inflation factor (VIF) <5 was considered to indicate no substantial multicollinearity. No substantial multicollinearity was identified among the explanatory variables included in the multivariable models, with all VIFs below 5 (range, 1.020-4.901) (**Supplementary Table 3**). To assess the robustness of the findings, sensitivity analyses were additionally conducted using a reduced set of variables. In a separate sensitivity analysis, the sPGA score was entered into the model as a categorical variable instead of a continuous variable. Regarding the analysis population, all 690 randomized patients were included. The present exploratory *post-hoc* analysis was based on the processed data set from the phase III study and therefore did not involve handling of missing data. In the original phase III study, missing data were handled using multiple imputation, with pooled estimates obtained according to Rubin’s rules; for other categorical efficacy endpoints, missing values were imputed as non-response, except for continuous variables, which were analyzed based on observed cases. All data processing was performed via SPSS version 29.0. A *P* < 0.05 indicated statistical significance.

## Results

### Study flow

A total of 1015 participants provided informed consent and were screened, of whom 690 were eligible and randomized in a 2:1 ratio to receive vunakizumab (N = 461) or placebo (N = 229). A total of 679 participants completed the 12-week induction treatment period, including 458 in the vunakizumab group and 221 in the placebo group. Subsequently, 677 participants entered the maintenance treatment period, of whom 576 completed the 40-week maintenance period, including 383 in the vunakizumab group and 193 in the placebo group. Finally, 586 participants completed the follow-up, including 389 in the vunakizumab group and 197 in the placebo group (**Supplementary Figure 1**).

### Clinical characteristics

The mean age was 41.3 ± 13.0 years in the enrolled patients. There were 522 (75.7%) males and 168 (24.3%) females. The mean BMI value was 25.3 ± 4.0 kg/m^2^. A total of 377 (54.6%) patients were non-smokers, and 313 (45.4%) patients were former or current smokers. There were 134 (19.4%) patients with a family history of psoriasis. One hundred and three (14.9%) patients had hypertension. The detailed clinical characteristics are shown in **Table 1**.

**Table 1:** Clinical characteristics of moderate-to-severe chronic plaque psoriasis patients.

Characteristics	Total patients	Any AE		*P* value
		Without (N = 208)	With (N = 482)	
Age (years), mean±SD	41.3±13.0	40.8±13.5	41.5±12.8	0.527^#^
Sex, n (%)				0.516^※^
Male	522 (75.7)	154 (74.0)	368 (76.3)	
Female	168 (24.3)	54 (26.0)	114 (23.7)	
BMI (kg/m^2^), mean±SD	25.3±4.0	24.8±3.6	25.6±4.1	0.013^#^
Smoking, n (%)				0.026^※^
Never	377 (54.6)	127 (61.1)	250 (51.9)	
Former or current	313 (45.4)	81 (38.9)	232 (48.1)	
Family history of psoriasis, n (%)				0.005^※^
No	556 (80.6)	181 (87.0)	375 (77.8)	
Yes	134 (19.4)	27 (13.0)	107 (22.2)	
Previous local treatment, n (%)				0.633^※^
No	137 (19.9)	39 (18.8)	98 (20.3)	
Yes	553 (80.1)	169 (81.3)	384 (79.7)	
Previous non-biological agent treatment, n (%)				0.208^※^
No	383 (55.5)	123 (59.1)	260 (53.9)	
Yes	307 (44.5)	85 (40.9)	222 (46.1)	
Previous biological agent treatment, n (%)				0.297^※^
No	658 (95.4)	201 (96.6)	457 (94.8)	
Yes	32 (4.6)	7 (3.4)	25 (5.2)	
Previous phototherapy, n (%)				0.444^※^
No	538 (78.0)	166 (79.8)	372 (77.2)	
Yes	152 (22.0)	42 (20.2)	110 (22.8)	
Hypertension, n (%)				0.035^※^
No	587 (85.1)	186 (89.4)	401 (83.2)	
Yes	103 (14.9)	22 (10.6)	81 (16.8)	
DM, n (%)				0.986^※^
No	660 (95.7)	199 (95.7)	461 (95.6)	
Yes	30 (4.3)	9 (4.3)	21 (4.4)	
Duration of psoriasis (years), median (IQR)	9.7 (4.2; 17.7)	9.6 (4.3; 16.8)	9.7 (4.1; 17.9)	0.953^*^
PASI score, median (IQR)	20.8 (15.2; 27.2)	20.2 (15.5; 27.3)	20.9 (15.0; 27.1)	0.765^*^
sPGA score, n (%)				0.008^*^
3	284 (41.2)	99 (47.6)	185 (38.4)	
4	344 (49.9)	98 (47.1)	246 (51.0)	
5	62 (9.0)	11 (5.3)	51 (10.6)	
BSA (%), median (IQR)	31.5 (21.5; 46.0)	32.6 (24.0; 44.0)	31.0 (20.0; 46.1)	0.139^*^
DLQI score, median (IQR)	10.0 (6.0; 16.0)	9.0 (5.0; 15.0)	11.0 (6.0; 16.0)	0.085^*^
Randomized treatment assignment, n (%)				0.478^※^
Vunakizumab	461 (66.8)	143 (68.8)	318 (66.0)	
Placebo	229 (33.2)	65 (31.3)	164 (34.0)	

AE, adverse event; SD, standard deviation; BMI, body mass index; DM, diabetes mellitus; IQR, interquartile range; PASI, psoriasis area and severity index; sPGA, static physician’ global assessment; BSA, body surface area; DLQI, dermatology life quality index.

Superscript ‘#’ indicates the *P* value was calculated by student *t* test; superscript ‘※’ indicates the *P* value was calculated by *Chi-square* test; superscript ‘*’ indicates the *P* value was calculated by Wilcoxon rank sum test.

A total of 461 (66.8%) patients received vunakizumab, and 229 (33.2%) patients received placebo. Baseline demographic and clinical characteristics were generally comparable between the vunakizumab and placebo groups, with no statistically significant differences observed in the assessed characteristics (all *P*>0.05) (**Supplementary Table 4**).

### Incidence of AEs

During the 12-week induction period, the incidence of any AE was 69.9% in all patients. A total of 366 (53.0%) patients experienced mild AEs, 110 (15.9%) patients experienced moderate AEs, and 6 (0.9%) patients experienced severe AEs. Seven (1.0%) patients experienced serious adverse events (SAEs), while treatment-related AEs occurred in 289 (41.9%) patients. AEs leading to treatment interruption and treatment discontinuation occurred in 9 (1.3%) and 4 (0.6%) patients, respectively. No deaths were reported during the 12-week induction period (**Supplementary Table 5**).

At the SOC level, the most frequent selected AE category was investigations (31.9%), followed by metabolic and nutritional disorders (26.7%), infectious and parasitic diseases (18.0%), and skin and subcutaneous tissue diseases (14.6%) (**Figure 1**).

**Figure 1:**
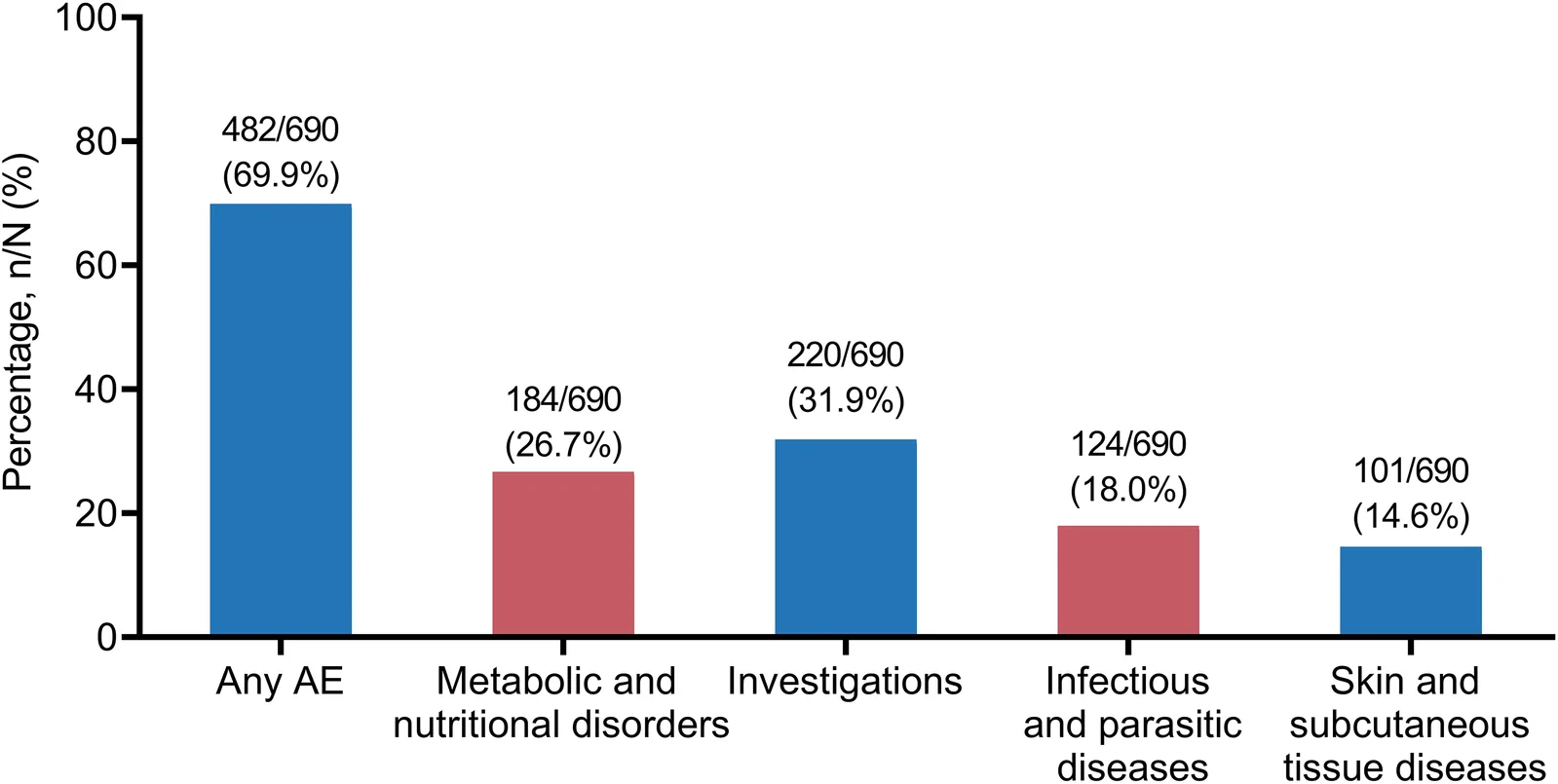
Incidence of any AE and selected AE categories at the SOC level during the 12-week induction period in all patients.

At the PT level, hyperuricemia, elevated liver enzymes, and injection site reaction were evaluated, with incidences of 11.3%, 7.1%, and 5.1%, respectively (**Supplementary Figure 2**).

Among the PTs within the skin and subcutaneous tissue diseases SOC category, the most frequently reported AEs were pruritus (5.7%), urticaria (2.3%), psoriasis (1.9%), and eczema (1.6%), followed by dermatitis allergic (0.9%), dermatitis atopic (0.7%), dry skin (0.7%), dermatitis (0.7%), alopecia (0.4%), acne (0.1%), erythema (0.1%), dermatitis contact (0.1%), and dermatitis seborrhoeic (0.1%) (**Supplementary Table 6**).

### Comparison of AEs between patients receiving vunakizumab and placebo

During the 12-week induction period, the incidence of any AE was not statistically different between the vunakizumab and placebo groups (RD: -2.6%; 95% CI: -9.8% to 4.6%). At the SOC level, the incidences of metabolic and nutritional disorders (RD: 1.4%; 95% CI: -5.6% to 8.3%), investigations (RD: 0.7%; 95% CI: -6.7% to 8.0%), and infectious and parasitic diseases (RD: -0.6%; 95% CI: -6.7% to 5.6%) also did not differ between the two groups. However, the incidence of skin and subcutaneous tissue diseases was lower in the vunakizumab group than in the placebo group (RD: -6.2%; 95% CI: -12.1% to -0.3%) (**Figure 2**). At the PT level, the incidences of elevated liver enzymes (RD: 2.8%; 95% CI, -1.0% to 6.6%) and hyperuricemia (RD: -2.7%; 95% CI: -7.9% to 2.5%) did not differ between the two groups, whereas the incidence of injection site reaction was higher in the vunakizumab group than in the placebo group (RD: 4.3%; 95% CI: 1.4% to 7.3%) (**Supplementary Figure 3**).

**Figure 2:**
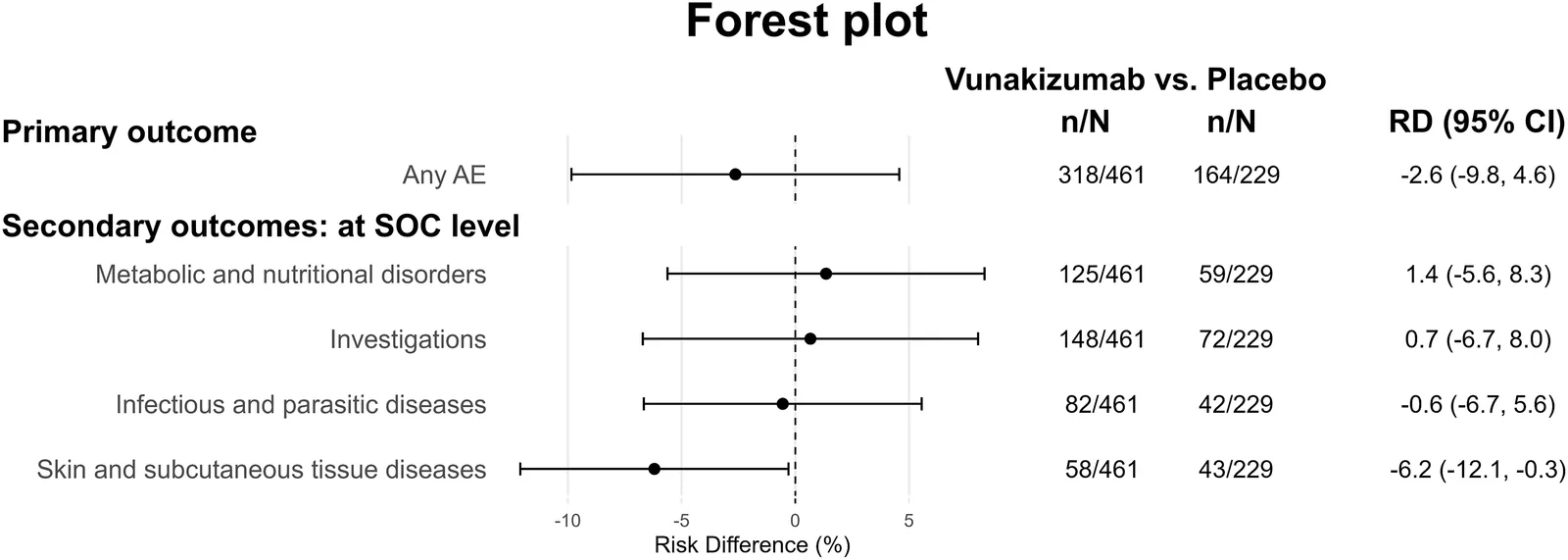
Comparison of the incidence of any AE and selected AE categories at the SOC level between the vunakizumab and placebo groups.

### Sensitivity analysis for any AE

BMI, smoking, family history of psoriasis, hypertension, and sPGA score were related to the occurrence of any AE in the univariable analyses (all *P* < 0.05). Further multivariable logistic regression analysis suggested that only the family history of psoriasis [odds ratio (OR): 2.015; 95% CI: 1.246 to 3.259; *P* = 0.004] and sPGA score (OR: 1.522; 95% CI: 1.076 to 2.154; *P* = 0.018) were independently related to the occurrence of any AE. Notably, the randomized treatment assignment (vunakizumab vs. placebo) was not an independent influencing factor for any AE (OR: 0.828; 95% CI: 0.574 to 1.193; *P* = 0.311). Apart from that, other factors were also not independently related to the occurrence of any AE, including age, sex, BMI, smoking, previous local treatment, previous non-biological agent treatment, previous biological agent treatment, previous phototherapy, hypertension, DM, duration of psoriasis, PASI score, BSA, and DLQI score (all *P*>0.05) (**Table 2**).

**Table 2:** Exploration of factors influencing any AE through logistic regression.

Factors	Univariable logistic regression analysis				Multivariable logistic regression analysis			
	*P* value	OR	95% CI		*P* value	OR	95% CI	
			Lower	Upper			Lower	Upper
Randomized treatment assignment (Vunakizumab vs. Placebo)	0.478	0.881	0.622	1.249	0.311	0.828	0.574	1.193
Age (per year)	0.527	1.004	0.992	1.017	0.686	1.003	0.988	1.018
Sex (Male vs. Female)	0.517	1.132	0.778	1.646	0.323	0.800	0.514	1.246
BMI (per kg/m^2^)	**0.014**	1.055	1.011	1.101	0.099	1.039	0.993	1.088
Smoking (Former or current vs. Never)	**0.026**	1.455	1.045	2.026	0.186	1.295	0.883	1.900
Family history of psoriasis (Yes vs. No)	**0.005**	1.913	1.210	3.024	**0.004**	2.015	1.246	3.259
Previous local treatment (Yes vs. No)	0.633	0.904	0.598	1.366	0.743	0.930	0.604	1.433
Previous non-biological agent treatment (Yes vs. No)	0.208	1.236	0.889	1.718	0.205	1.252	0.885	1.771
Previous biological agent treatment (Yes vs. No)	0.300	1.571	0.668	3.691	0.287	1.618	0.668	3.920
Previous phototherapy (Yes vs. No)	0.445	1.169	0.784	1.743	0.590	1.121	0.741	1.695
Hypertension (Yes vs. No)	**0.037**	1.708	1.033	2.822	0.060	1.706	0.977	2.981
DM (Yes vs. No)	0.986	1.007	0.453	2.238	0.529	0.760	0.323	1.788
Duration of psoriasis (per year)	0.701	0.997	0.980	1.014	0.268	0.989	0.970	1.009
PASI score (per score)	0.813	1.002	0.985	1.019	0.883	0.997	0.960	1.036
sPGA score (per score)	**0.006**	1.455	1.115	1.898	**0.018**	1.522	1.076	2.154
BSA (per %)	0.466	0.997	0.988	1.006	0.495	0.994	0.976	1.012
DLQI score (per score)	0.168	1.016	0.993	1.040	0.432	1.010	0.986	1.034

AE, adverse event; OR, odds ratio; CI, confidence interval; BMI, body mass index; DM, diabetes mellitus; PASI, psoriasis area and severity index; sPGA, static physician's global assessment; BSA, body surface area; DLQI, dermatology life quality index.

The sPGA score was additionally categorized, with an sPGA score of 3 used as the reference category, and the multivariable logistic regression analysis was repeated. The results were generally consistent with those of the original analysis. Family history of psoriasis remained independently associated with a higher likelihood of any AE (OR: 2.002; 95% CI: 1.238 to 3.239; *P* = 0.005), and an sPGA score of 5 was independently associated with a higher likelihood of any AE compared with an sPGA score of 3 (OR: 2.902; 95% CI: 1.222 to 6.893; *P* = 0.016). Vunakizumab was not significantly associated with the occurrence of any AE (OR: 0.825; 95% CI: 0.572 to 1.189; *P* = 0.302) (**Supplementary Table 7**).

### Sensitivity analysis for selected AE categories

According to the univariable logistic regression analysis, vunakizumab was associated with a lower likelihood of skin and subcutaneous tissue diseases (OR: 0.623; 95% CI: 0.405 to 0.958; *P* = 0.031) at the SOC level. At the PT level, vunakizumab was associated with a higher likelihood of injection site reaction (OR: 3.118; 95% CI: 1.193 to 8.148; *P* = 0.020). In the multivariable logistic regression analysis, vunakizumab remained independently associated with a lower likelihood of skin and subcutaneous tissue diseases (OR: 0.575; 95% CI: 0.367 to 0.903; *P* = 0.016) at the SOC level. At the PT level, vunakizumab was independently associated with a higher likelihood of injection site reaction (OR: 2.986; 95% CI: 1.118 to 7.975; *P* = 0.029). No significant independent associations were observed for the other selected AE categories at either the SOC or PT level (all *P*>0.05) (**Table 3**).

**Table 3:** Effects of vunakizumab on selected AE categories: a univariable and multivariable logistic regression analysis.

Items	Univariable logistic regression analysis				Multivariable logistic regression analysis			
	*P* value	OR	95% CI		*P* value	OR	95% CI	
			Lower	Upper			Lower	Upper
SOC level								
Metabolic and nutritional disorders	0.706	1.072	0.748	1.537	0.413	1.173	0.801	1.717
Investigations	0.860	1.031	0.733	1.450	0.604	0.908	0.630	1.308
Infectious and parasitic diseases	0.859	0.963	0.639	1.453	0.675	0.913	0.595	1.400
Skin and subcutaneous tissue diseases	**0.031**	0.623	0.405	0.958	**0.016**	0.575	0.367	0.903
PT level								
Elevated liver enzymes	0.183	1.578	0.806	3.088	0.333	1.415	0.701	2.857
Hyperuricemia	0.295	0.771	0.474	1.254	0.409	0.806	0.484	1.344
Injection site reaction	**0.020**	3.118	1.193	8.148	**0.029**	2.986	1.118	7.975

AE, adverse event; PT, preferred term; SOC, system organ classes; OR, odds ratio; CI, confidence interval. Only the regression estimates for randomized treatment assignment (Vunakizumab vs. Placebo) from each univariable and multivariable logistic regression model are presented. Randomized treatment assignment (Vunakizumab vs. Placebo) in the multivariable logistic regression analysis was adjusted for Age (per year), Sex (Male vs. Female), Body mass index (per kg/m^2^), Smoking (Former or current vs. Never), Family history of psoriasis (Yes vs. No), Previous local treatment (Yes vs. No), Previous non-biological agent treatment (Yes vs. No), Previous biological agent treatment (Yes vs. No), Previous phototherapy (Yes vs. No), Hypertension (Yes vs. No), Diabetes mellitus (Yes vs. No), Duration of psoriasis (per year), Psoriasis area and severity index score (per score), Static physician's global assessment score (per score), body surface area (per %), Dermatology life quality index score (per score).

Sensitivity analyses were further performed by categorizing the sPGA score and using a reduced covariate set. After categorizing the sPGA score, at the SOC level, the association between vunakizumab and skin and subcutaneous tissue diseases remained significant in Model 1, which included psoriasis exacerbation (OR: 0.575; 95% CI: 0.367 to 0.902; *P* = 0.016). At the PT level, vunakizumab remained significantly associated with a higher likelihood of injection site reaction (OR: 2.999; 95% CI: 1.122 to 8.014; *P* = 0.029). Using the reduced covariate set, vunakizumab remained significantly associated with a lower likelihood of skin and subcutaneous tissue diseases in Model 1 (OR: 0.600; 95% CI: 0.387 to 0.930; *P* = 0.022) and a higher likelihood of injection site reaction (OR: 3.117; 95% CI: 1.188 to 8.180; *P* = 0.021). Notably, when psoriasis exacerbation was excluded from the skin and subcutaneous tissue diseases outcome (Model 2), the association was no longer statistically significant after either categorizing the sPGA score (OR: 0.810; 95% CI: 0.500 to 1.311; *P* = 0.391) or using the reduced covariate set (OR: 0.851; 95% CI: 0.531 to 1.364; *P* = 0.503) (**Supplementary Table 8**).

## Discussion

In this exploratory *post-hoc* analysis, several interesting findings were discovered (1). No statistically significant difference in any AE was observed between the treatment groups during the 12-week induction period, while a family history of psoriasis and baseline sPGA score were two independent risk factors for any AEs (2). The incidence of selected AE categories during the 12-week induction period, including metabolic and nutritional disorders, investigations, infectious and parasitic diseases, elevated liver enzymes, and hyperuricemia, was not statistically different between the vunakizumab and placebo groups (3). Vunakizumab was associated with a lower likelihood of skin and subcutaneous tissue diseases at the SOC level and a higher likelihood of injection site reaction at the PT level. However, the association of vunakizumab with skin and subcutaneous tissue diseases was no longer significant after excluding psoriasis exacerbation.

IL-17 inhibitors, such as secukinumab and brodalumab, show good safety profiles in patients with moderate-to-severe plaque psoriasis (12–14). As reported by previous studies, the occurrence rates of AEs during the induction period were similar between several IL-17 inhibitors and placebo in patients with moderate-to-severe plaque psoriasis (15–18). In line with the findings of these previous studies, we also observed that the occurrence of any AE during the induction period was not different between vunakizumab and placebo in patients with moderate-to-severe plaque psoriasis. Our multivariable logistic regression analysis further verified this finding. Family history of psoriasis and higher baseline sPGA score were independently associated with a higher likelihood of any AE. However, these associations should be considered exploratory. Further studies in independent populations are needed to validate these findings and determine whether these associations are reproducible.

Patients with moderate-to-severe plaque psoriasis often experience nasopharyngitis, oral candidiasis, urinary tract infection, upper respiratory tract infection, injection site reaction, headache, diarrhea, and pruritus following the administration of IL-17 inhibitors (19–23). In this exploratory *post-hoc* analysis, seven selected AE categories were extracted from the previous phase III trial to investigate the impact of vunakizumab on these AEs, including four categories at the SOC level (investigations, metabolic and nutritional disorders, infectious and parasitic diseases, and skin and subcutaneous tissue diseases) and three at the PT level (elevated liver enzymes, hyperuricemia, and injection site reaction). Among these selected AE categories, metabolic and nutritional disorders frequently occurred in patients with moderate-to-severe plaque psoriasis according to the previous phase III trial (11). Additionally, this AE is also of particular concern to clinicians. Surprisingly, at the SOC level, we found that the incidence of skin and subcutaneous tissue diseases was significantly lower in patients receiving vunakizumab than in those receiving placebo. However, sensitivity analysis showed that this association was no longer significant after excluding psoriasis exacerbation, suggesting that the observed difference may largely reflect improved control of psoriasis rather than a specific safety benefit of vunakizumab. In contrast, at the PT level, the incidence of injection site reaction was significantly higher in patients receiving vunakizumab than in those receiving placebo. This finding was consistent with the known local tolerability profile of subcutaneously administered biologic therapies, as injection site reactions may occur following subcutaneous administration (24–26). The incidence of other selected AE categories was not different between patients receiving vunakizumab and those receiving placebo.

In the original phase III trial, hyperlipidemia (7.8% vs. 6.1%) and hypertriglyceridemia (6.7% vs. 5.2%) during the induction period appeared to occur more frequently in patients receiving vunakizumab than in those receiving placebo (11). However, in the present exploratory *post-hoc* analysis, no significant association was observed between the treatments and the occurrence of metabolic and nutritional disorders. This apparent discrepancy may be related to the differences in the analytical approach, as the present exploratory *post-hoc* analysis evaluated the broader category of metabolic and nutritional disorders rather than individual metabolic events. Given that the baseline characteristics were generally well balanced between the treatment groups in the original randomized trial, substantial baseline confounding is unlikely to account for this discrepancy between the findings of the original trial and the present exploratory *post-hoc* analysis.

Compared with the original phase III trial, the present exploratory *post-hoc* analysis provides a more detailed characterization of selected AEs by evaluating them at both the SOC and PT levels and exploring potential factors associated with AE occurrence using multivariable logistic regression. These additional analyses provide hypothesis-generating information on potential baseline factors associated with AE occurrence and help further characterize the safety profile of vunakizumab beyond the overall AE incidence reported in the original trial. Further studies in independent populations are needed to validate these findings and determine whether these associations are reproducible.

Several limitations could not be omitted in this study (1). All data were derived from the previous trial. Therefore, the extracted clinical characteristics might not fully cover the potential risk factors for AEs. Further studies should consider including as many clinical characteristics as possible to comprehensively explore the risk factors for AEs (2). This was an exploratory *post-hoc* exploratory analysis. Therefore, the observed associations should be interpreted cautiously as hypothesis-generating findings (3). Multiple comparisons may have increased the possibility of chance findings (4). The placebo-controlled period was relatively short, and the long-term safety profile of vunakizumab requires further evaluation (5). Given the low incidence of some AEs, the present analysis had limited ability to evaluate rare events reliably (6). No active comparator was included in the original trial; therefore, the safety findings of vunakizumab could not be directly compared with those of other active treatments.

## Conclusion

In summary, vunakizumab is not associated with the occurrence of any AE or most selected AE categories in patients with moderate-to-severe plaque psoriasis. However, vunakizumab is associated with a higher likelihood of injection site reaction and a lower likelihood of skin and subcutaneous tissue diseases. The observed lower incidence of skin and subcutaneous tissue diseases may be largely related to improved control of psoriasis by vunakizumab.

## Data Availability

The original contributions presented in the study are included in the article/**Supplementary Material**. Further inquiries can be directed to the corresponding authors.

## Supplementary material

Supplementary Figure 1 CONSORT flow diagram of participant disposition in the original phase III trial.

Supplementary Figure 2 Incidence of selected AE categories at the PT level during the 12-week induction period in all patients.

Supplementary Figure 3 Comparison of the incidence of selected AE categories at the PT level between the vunakizumab and placebo groups.
