# Supplementary material for: Selected adverse events during the 12-week induction period of a phase III trial of vunakizumab: an exploratory post-hoc analysis
Source: Front Immunol. 2026 Oct 14;17:1894231. doi: 10.3389/fimmu.2026.1894231 (PMC13713518; doi:10.3389/fimmu.2026.1894231)
Supplement: Supplementary file 1 [file Table1.docx]

**Supplementary Table 1.** Participated centers.

| **Trial site** | **Ethics approval number** |
| --- | --- |
| Huashan Hospital, Fudan University | 20201231 |
| Dermatology Hospital of Southern Medical University | Approved (filed) |
| Nanfang Hospital, Southern Medical University | NFEC-202104-Y13 |
| Guangdong Provincial Hospital of Chinese Medicine | AF2021-090-02 |
| The Fourth Hospital of Hebei Medical University | 2021013-3 |
| Peking University Third Hospital | 2021-046-06 |
| The First Affiliated Hospital of Xi’an Jiaotong University | XJTUIAF2021LSY-90-4 |
| Nanyang First People’s Hospital | 20210005-09 |
| The Second Affiliated Hospital of Kunming Medical University | YJ-2023-32 |
| The First Affiliated Hospital of Kunming Medical University | 2021-9-8 |
| Beijing Chaoyang Hospital, Capital Medical University | Approved (filed) |
| The Second Hospital of Hebei Medical University | 2021EC03-04-XZ-4 |
| The Second Hospital of Dalian Medical University | 2021-091 |
| Beijing Friendship Hospital, Capital Medical University | 2021-P1-013-07 |
| Shiyan Renmin Hospital | Approved (filed) |
| Renmin Hospital of Wuhan University | WDRY2021-Y061（X03） |
| The First Affiliated Hospital of Guangxi Medical University | 2022-294 |
| Tianjin Academy of Traditional Chinese Medicine Affiliated Hospital | LLSY2021-08-03 |
| Union Hospital, Tongji Medical College, Huazhong University of Science and Technology | 2021-0147-07 |
| Suining Central Hospital | 2021-sy002-12 |
| Wuxi No. 2 People’s Hospital | 2023-2023011 |
| Jiangsu Province Hospital | 2021-MD-125.A1 |
| Hospital for Skin Diseases, Chinese Academy of Medical Sciences | 2021-005 |
| The First Affiliated Hospital of Fujian Medical University | LY2021-012-12 |
| The First Bethune Hospital of Jilin University | 21Y064-008 |
| The Second Norman Bethune Hospital of Jilin University | 2021-15-3 |
| The First Affiliated Hospital of Wenzhou Medical University | 2021-025-X03 |
| Peking University First Hospital | 2021-061-003 |
| Hangzhou Third People’s Hospital | 2022-071 |
| The Affiliated Hospital of Guizhou Medical University | 2021034-AMD-09 |
| The First Affiliated Hospital of Chongqing Medical University | CY2021-S3-04 |
| The Southwest Hospital of Army Medical University | BAYW202208 |
| The Second Affiliated Hospital of Nanchang University | 2021-26-04 |
| The First Affiliated Hospital of Nanchang University | 2021-082-4 |
| The First Affiliated Hospital of China Medical University | 2021YL068-4 |
| Sir Run Run Shaw Hospital, Zhejiang University School of Medicine | 2023-0251 |
| West China Hospital, Sichuan University | Approved (filed) |
| The Second Hospital of Anhui Medical University | YJ-YW2021-045-F1-4 |
| Anhui Provincial Hospital | 2022-614 |
| The Third Xiangya Hospital of Central South University | 22542 |
| Chongqing Traditional Chinese Medicine Hospital | 2021-4 |
| Henan Provincial People’s Hospital | 2021-026-10 |
| Yueyang Hospital of Integrated Traditional Chinese and Western Medicine, Shanghai University of Traditional Chinese Medicine | 2021-063-09 |
| Shanghai Skin Disease Hospital | 2021-09-X4 |
| Peking Union Medical College Hospital | KS2023089 |
| Shengjing Hospital of China Medical University | 2021PS034 |
| The First Affiliated Hospital of Anhui Medical University | PJ2021-06-07 (1) |
| The Second Affiliated Hospital of Air Force Military Medical University | K202105-01 |
