# Supplementary material for: Selected adverse events during the 12-week induction period of a phase III trial of vunakizumab: an exploratory post-hoc analysis
Source: Front Immunol. 2026 Oct 14;17:1894231. doi: 10.3389/fimmu.2026.1894231 (PMC13713518; doi:10.3389/fimmu.2026.1894231)
Supplement: Supplementary file 2 [file Table2.docx]

**Supplementary Table 2.** PTs in each SOC category.

| SOC | PTs |
| --- | --- |
| Metabolic and nutritional disorders | Diabetes mellitus, Hypokalaemia, Hyperglycaemia, Hypophosphataemia, Hyperlipidaemia, Hyperuricaemia, Hyponatraemia, Hypertriglyceridaemia, Hypercholesterolaemia, Electrolyte imbalance, Hyperhomocysteinaemia, Type 2 diabetes mellitus, Dyslipidaemia, Hypocalcaemia, Lipid metabolism disorder, Decreased appetite, Hypoproteinaemia, Hyperphosphataemia, Hyperkalaemia, Vitamin D deficiency, Hypoalbuminaemia, Impaired glucose tolerance, Gout |
| Investigations | Monocyte percentage increased, Low density lipoprotein increased, Non-high density lipoprotein cholesterol increased, Lymphocyte percentage decreased, Lymphocyte count increased, Eosinophil percentage increased, Eosinophil count increased, Glycosylated haemoglobin increased, Blood glucose increased, Blood lactate dehydrogenase increased, Blood triglycerides increased, Monocyte count increased, Platelet count decreased, White blood cell count increased, Alanine aminotransferase increased, Non-high density lipoprotein cholesterol, Lymphocyte percentage increased, Weight increased, Aspartate aminotransferase increased, Electrocardiogram T wave abnormal, Blood cholesterol increased, Blood alkaline phosphatase increased, Blood pressure increased, High density lipoprotein decreased, Blood uric acid increased, White blood cell count decreased, Lymphocyte count decreased, Neutrophil count decreased, Blood chloride decreased, Neutrophil percentage increased, Neutrophil count increased, Neutrophil percentage decreased, Blood magnesium increased, Platelet count increased, Basophil percentage increased, Blood potassium decreased, Mononuclear cell count increased, Antinuclear antibody positive, Blood bilirubin increased, Blood urine present, Electrocardiogram T wave amplitude decreased, Blood phosphorus decreased, Antinuclear antibody increased, Electrocardiogram U wave abnormal, Blood creatinine increased, Occult blood positive, Electrocardiogram ST-T segment change, Urine occult blood positive, Interferon gamma release assay positive, Weight decreased, Gamma-glutamyltransferase increased, Blood creatine phosphokinase increased, White blood cells urine positive, Protein urine present, Red blood cells urine positive, Crystals urine present, Hepatitis B surface antigen positive, Electrocardiogram QRS axis abnormal, Bacterial test positive, SARS-CoV-2 test positive, Bilirubin urine present, Bilirubin urine increased, Ketone bodies urine present, Lipids increased, Hepatic enzyme increased, Treponema test positive, Carotid intima-media thickness increased, Transaminases increased, Electrocardiogram ST segment elevation, Electrocardiogram T wave peaked, Blood cholinesterase increased, Electrocardiogram voltage increased, Total bile acids increased, Glucose urine present, White blood cell differential count abnormal, Lipoprotein increased, Blood bilirubin unconjugated increased, C-reactive protein increased, Basophil count increased, Myocardial necrosis marker increased, Parietal cell antibody positive, Alpha-hydroxybutyrate dehydrogenase increased, Fibrin D dimer increased, Blood urea increased, Breast ultrasound abnormal, Haemoglobin decreased, Hepatic computed tomography abnormal, Electrocardiogram ST segment abnormal, Hepatitis B e antibody positive, Hepatitis B core antibody positive, Electrocardiogram T wave inversion, Urine casts present, Electrocardiogram Q wave abnormal, Glomerular filtration rate decreased, Electrocardiogram QT prolonged, Bilirubin conjugated increased, Blood creatine phosphokinase MB increased, Blood potassium increased, Electrocardiogram PR prolongation, Red blood cell count increased, Urological examination abnormal, Blood ketone body increased, Mycobacterium tuberculosis complex test positive, Free fatty acids increased, Amylase increased, Red blood cell sedimentation rate increased, Electrocardiogram abnormal, Mononuclear cell count decreased, Double stranded DNA antibody positive, Cystatin C increased, Urine albumin positive, Urine specific gravity increased, Urine sediment present, Blood homocysteine increased, Electrocardiogram repolarisation abnormality, Renal ultrasound abnormal, Urobilinogen urine increased |
| Infectious and parasitic diseases | Tinea pedis, Upper respiratory tract infection, Onychomycosis, Tonsillitis, Rhinitis, Hordeolum, Otitis media, Folliculitis, Fungal skin infection, Pharyngitis, Urinary tract infection, Infected dermal cyst, Conjunctivitis, Fungal infection, Skin infection, Gastroenteritis, Nasopharyngitis, Otitis externa, Pharyngolaryngitis, Tinea manuum, Paronychia, Hepatitis B, Bronchitis, Respiratory tract infection, COVID-19, Suspected COVID-19, Acute sinusitis, Pneumonia, COVID-19 pneumonia, Furuncle, Scrotal infection, Herpes zoster, Eczema infected, Viral infection, Laryngitis, Omphalitis, Urethritis, Influenza, Helicobacter infection, Pulpitis, Sinusitis, Herpes simplex, Cervicitis, Tinea infection, Otitis externa fungal, Pityriasis versicolor, Tinea corporis, Mastitis, Tracheobronchitis, Myringitis, Latent tuberculosis, Pericoronitis, Periodontitis, Hepatitis C, Lip infection, Genital herpes, Gastroenteritis viral, Chronic hepatitis B, Tonsillitis bacterial, Oral herpes, Vaginal infection, Soft tissue infection, Pneumonia bacterial, Genital infection, Tinea cruris, Wound infection, Ureteritis, Gingivitis, Hepatitis virus carrier, Papilloma virus infection, Pyelonephritis, Acute pyelonephritis, Bacterial infection, Infection |
| Skin and subcutaneous tissue diseases | Rash, Dermatitis allergic, Urticaria, Erythema, Pruritus, Eczema, Dermatitis atopic, Acne, Seborrhoeic dermatitis, Psoriasis, Dermatitis, Dry skin, Skin exfoliation, Alopecia areata, Dermatitis contact, Hand dermatitis, Alopecia, Hypertrichosis, Yellow skin, Enlarged skin pores, Angioedema, Hyperhidrosis, Photosensitivity reaction, Rosacea, Cold sweat, Nail disorder, Ecchymosis, Papular urticaria, Dyshidrotic eczema, Miliaria, Pityriasis rosea, Hair texture abnormal, Skin lesion, Blister, Rash pruritic, Urticaria physical, Neurodermatitis, Dermal cyst, Keloid, Lichen striatus, Pain of skin |

SOC, system organ classes; PTs, preferred terms.
