# Supplementary material for: Selected adverse events during the 12-week induction period of a phase III trial of vunakizumab: an exploratory post-hoc analysis
Source: Front Immunol. 2026 Oct 14;17:1894231. doi: 10.3389/fimmu.2026.1894231 (PMC13713518; doi:10.3389/fimmu.2026.1894231)
Supplement: Supplementary file 3 [file Table3.docx]

**Supplementary Table 3.** Multicollinearity diagnostics for variables included in the multivariable model.

| Factors | Tolerance | VIF |
| --- | --- | --- |
|  |  |  |
| Randomized treatment assignment (Vunakizumab vs. Placebo) | 0.962 | 1.039 |
| Age | 0.725 | 1.379 |
| Sex | 0.753 | 1.329 |
| BMI | 0.920 | 1.086 |
| Smoking | 0.765 | 1.307 |
| Family history of psoriasis | 0.953 | 1.050 |
| Previous local treatment | 0.970 | 1.031 |
| Previous non-biological agent treatment | 0.952 | 1.050 |
| Previous biological agent treatment | 0.955 | 1.047 |
| Previous phototherapy | 0.980 | 1.020 |
| Hypertension | 0.827 | 1.209 |
| DM | 0.934 | 1.071 |
| Duration of psoriasis | 0.794 | 1.259 |
| PASI score | 0.204 | 4.901 |
| sPGA score | 0.610 | 1.638 |
| BSA | 0.265 | 3.776 |
| DLQI score | 0.960 | 1.042 |

VIF, variance inflation factor; BMI, body mass index; DM, diabetes mellitus; PASI, psoriasis area and severity index; sPGA, static physician’ global assessment; BSA, body surface area; DLQI, dermatology life quality index.

A VIF <5 was considered to indicate no substantial multicollinearity among the included variables.
