# Supplementary material for: Selected adverse events during the 12-week induction period of a phase III trial of vunakizumab: an exploratory post-hoc analysis
Source: Front Immunol. 2026 Oct 14;17:1894231. doi: 10.3389/fimmu.2026.1894231 (PMC13713518; doi:10.3389/fimmu.2026.1894231)
Supplement: Supplementary file 4 [file Table4.docx]

**Supplementary Table 4.** Clinical characteristics of patients in the vunakizumab and placebo groups.

| Characteristics | Vunakizumab  (N = 461) | Placebo  (N = 229) | *P* value |
| --- | --- | --- | --- |
| Age (years), mean±SD | 41.7±13.2 | 40.4±12.6 | 0.227^#^ |
| Sex, n (%) |  |  | 0.541^※^ |
| Male | 352 (76.4) | 170 (74.2) |  |
| Female | 109 (23.6) | 59 (25.8) |  |
| BMI (kg/m^2^), mean±SD | 25.4±3.9 | 25.3±4.0 | 0.711^#^ |
| Smoking, n (%) |  |  | 0.640^※^ |
| Never | 249 (54.0) | 128 (55.9) |  |
| Former or current | 212 (46.0) | 101 (44.1) |  |
| Family history of psoriasis, n (%) |  |  | 0.478^※^ |
| No | 368 (79.8) | 188 (82.1) |  |
| Yes | 93 (20.2) | 41 (17.9) |  |
| Previous local treatment, n (%) |  |  | 0.474^※^ |
| No | 88 (19.1) | 49 (21.4) |  |
| Yes | 373 (80.9) | 180 (78.6) |  |
| Previous non-biological agent treatment, n (%) |  |  | 0.071^※^ |
| No | 267 (57.9) | 116 (50.7) |  |
| Yes | 194 (42.1) | 113 (49.3) |  |
| Previous biological agent treatment, n (%) |  |  | 0.533^※^ |
| No | 438 (95.0) | 220 (96.1) |  |
| Yes | 23 (5.0) | 9 (3.9) |  |
| Previous phototherapy, n (%) |  |  | 0.778^※^ |
| No | 358 (77.7) | 180 (78.6) |  |
| Yes | 103 (22.3) | 49 (21.4) |  |
| Hypertension, n (%) |  |  | 0.240^※^ |
| No | 387 (83.9) | 200 (87.3) |  |
| Yes | 74 (16.1) | 29 (12.7) |  |
| DM, n (%) |  |  | 0.438^※^ |
| No | 439 (95.2) | 221 (96.5) |  |
| Yes | 22 (4.8) | 8 (3.5) |  |
| Duration of psoriasis (years), median (IQR) | 9.5 (4.1; 16.5) | 10.1 (4.6; 19.3) | 0.191^*^ |
| PASI score, median (IQR) | 20.1 (15.0; 26.4) | 21.3 (15.5; 29.3) | 0.139^*^ |
| sPGA score, n (%) |  |  | 0.764^*^ |
| 3 | 188 (40.8) | 96 (41.9) |  |
| 4 | 231 (50.1) | 113 (49.3) |  |
| 5 | 42 (9.1) | 20 (8.7) |  |
| BSA (%), median (IQR) | 31.0 (21.8; 44.7) | 32.0 (21.0; 47.5) | 0.413^*^ |
| DLQI score, median (IQR) | 10.0 (6.0; 15.0) | 11.0 (6.00; 17.0) | 0.186^*^ |

SD, standard deviation; BMI, body mass index; DM, diabetes mellitus; IQR, interquartile range; PASI, psoriasis area and severity index; sPGA, static physician’ global assessment; BSA, body surface area; DLQI, dermatology life quality index.

Superscript ‘#’ indicates the *P* value was calculated by student *t* test; superscript ‘※’ indicates the *P* value was calculated by *Chi-square* test; superscript ‘*’ indicates the *P* value was calculated by Wilcoxon rank sum test.
