# Supplementary material for: Selected adverse events during the 12-week induction period of a phase III trial of vunakizumab: an exploratory post-hoc analysis
Source: Front Immunol. 2026 Oct 14;17:1894231. doi: 10.3389/fimmu.2026.1894231 (PMC13713518; doi:10.3389/fimmu.2026.1894231)
Supplement: Supplementary file 5 [file Table5.docx]

**Supplementary Table 5.** AEs during the 12-week induction period.

| Items | Patients (N = 690) |
| --- | --- |
| Any AE, n (%) | 482 (69.9) |
| Mild intensity | 366 (53.0) |
| Moderate intensity | 110 (15.9) |
| Severe intensity | 6 (0.9) |
| SAE, n (%) | 7 (1.0) |
| Treatment-related AEs, n (%) | 289 (41.9) |
| AE leading to treatment interruption, n (%) | 9 (1.3) |
| AE leading to treatment discontinuation, n (%) | 4 (0.6) |
| Death, n (%) | 0 (0.0) |

AE, adverse event; SAE, serious adverse event.
