# Supplementary material for: Selected adverse events during the 12-week induction period of a phase III trial of vunakizumab: an exploratory post-hoc analysis
Source: Front Immunol. 2026 Oct 14;17:1894231. doi: 10.3389/fimmu.2026.1894231 (PMC13713518; doi:10.3389/fimmu.2026.1894231)
Supplement: Supplementary file 6 [file Table6.docx]

**Supplementary Table 6.** PTs in skin and subcutaneous tissue diseases SOC category.

| Items | Patients (N = 690) |
| --- | --- |
| Pruritus, n (%) | 39 (5.7) |
| Urticaria, n (%) | 16 (2.3) |
| Psoriasis, n (%) | 13 (1.9) |
| Eczema, n (%) | 11 (1.6) |
| Dermatitis atopic, n (%) | 5 (0.7) |
| Alopecia, n (%) | 3 (0.4) |
| Dermatitis allergic, n (%) | 6 (0.9) |
| Dry skin, n (%) | 5 (0.7) |
| Dermatitis, n (%) | 5 (0.7) |
| Acne, n (%) | 1 (0.1) |
| Erythema, n (%) | 1 (0.1) |
| Dermatitis contact, n (%) | 1 (0.1) |
| Dermatitis seborrhoeic, n (%) | 1 (0.1) |

PTs, preferred terms; SOC, system organ classes.
