# Supplementary material for: Selected adverse events during the 12-week induction period of a phase III trial of vunakizumab: an exploratory post-hoc analysis
Source: Front Immunol. 2026 Oct 14;17:1894231. doi: 10.3389/fimmu.2026.1894231 (PMC13713518; doi:10.3389/fimmu.2026.1894231)
Supplement: Supplementary file 7 [file Table7.docx]

**Supplementary Table 7.** Sensitivity analysis for any AE after categorizing sPGA score.

| Factors | *P* value | OR | 95% CI | |
| --- | --- | --- | --- | --- |
|  |  |  | Lower | Upper |
| Randomized treatment assignment (Vunakizumab vs. Placebo) | 0.302 | 0.825 | 0.572 | 1.189 |
| Age (per year) | 0.695 | 1.003 | 0.988 | 1.018 |
| Sex (Male vs. Female) | 0.304 | 0.793 | 0.509 | 1.235 |
| BMI (per kg/m^2^) | 0.089 | 1.041 | 0.994 | 1.090 |
| Smoking (Former or current vs. Never) | 0.175 | 1.305 | 0.889 | 1.915 |
| Family history of psoriasis (Yes vs. No) | **0.005** | 2.002 | 1.238 | 3.239 |
| Previous local treatment (Yes vs. No) | 0.746 | 0.931 | 0.604 | 1.435 |
| Previous non-biological agent treatment (Yes vs. No) | 0.179 | 1.270 | 0.896 | 1.800 |
| Previous biological agent treatment (Yes vs. No) | 0.282 | 1.626 | 0.670 | 3.942 |
| Previous phototherapy (Yes vs. No) | 0.623 | 1.110 | 0.733 | 1.681 |
| Hypertension (Yes vs. No) | 0.063 | 1.698 | 0.972 | 2.969 |
| DM (Yes vs. No) | 0.533 | 0.761 | 0.323 | 1.795 |
| Duration of psoriasis (per year) | 0.275 | 0.989 | 0.970 | 1.009 |
| PASI score (per score) | 0.802 | 0.995 | 0.957 | 1.034 |
| sPGA score (3 as reference) | (-) | 1.000 | (-) | (-) |
| 4 vs. 3 | 0.103 | 1.392 | 0.935 | 2.071 |
| 5 vs. 3 | **0.016** | 2.902 | 1.222 | 6.893 |
| BSA (per %) | 0.539 | 0.994 | 0.976 | 1.013 |
| DLQI score (per score) | 0.486 | 1.009 | 0.985 | 1.033 |

AE, adverse event; OR, odds ratio; CI, confidence interval; BMI, body mass index; DM, diabetes mellitus; PASI, psoriasis area and severity index; sPGA, static physician's global assessment; BSA, body surface area; DLQI, dermatology life quality index.
