# Supplementary material for: Selected adverse events during the 12-week induction period of a phase III trial of vunakizumab: an exploratory post-hoc analysis
Source: Front Immunol. 2026 Oct 14;17:1894231. doi: 10.3389/fimmu.2026.1894231 (PMC13713518; doi:10.3389/fimmu.2026.1894231)
Supplement: Supplementary file 8 [file Table8.docx]

**Supplementary Table 8.** Sensitivity analysis of the associations between vunakizumab and selected AE categories.

| Items | After categorizing sPGA score^*^ | | | |  | Reduced covariate set^#^ | | | |
| --- | --- | --- | --- | --- | --- | --- | --- | --- | --- |
|  | *P* value | OR | 95% CI | |  | *P* value | OR | 95% CI | |
|  |  |  | Lower | Upper |  |  |  | Lower | Upper |
| **SOC level** |  |  |  |  |  |  |  |  |  |
| Metabolic and nutritional disorders | 0.413 | 1.173 | 0.801 | 1.716 |  | 0.633 | 1.093 | 0.760 | 1.570 |
| Investigations | 0.598 | 0.906 | 0.629 | 1.306 |  | 0.950 | 1.011 | 0.712 | 1.436 |
| Infectious and parasitic diseases | 0.675 | 0.913 | 0.595 | 1.400 |  | 0.970 | 0.992 | 0.654 | 1.505 |
| Skin and subcutaneous tissue diseases^※^ |  |  |  |  |  |  |  |  |  |
| Model 1 | **0.016** | 0.575 | 0.367 | 0.902 |  | **0.022** | 0.600 | 0.387 | 0.930 |
| Model 2 | 0.391 | 0.810 | 0.500 | 1.311 |  | 0.503 | 0.851 | 0.531 | 1.364 |
| **PT level** |  |  |  |  |  |  |  |  |  |
| Elevated liver enzymes | 0.341 | 1.409 | 0.696 | 2.853 |  | 0.218 | 1.533 | 0.777 | 3.025 |
| Hyperuricemia | 0.406 | 0.805 | 0.483 | 1.342 |  | 0.322 | 0.781 | 0.479 | 1.274 |
| Injection site reaction | **0.029** | 2.999 | 1.122 | 8.014 |  | **0.021** | 3.117 | 1.188 | 8.180 |

AE, adverse event; PT, preferred term; SOC, system organ classes; sPGA, Static physician's global assessment score; OR, odds ratio; CI, confidence interval.

*, Randomized treatment assignment (Vunakizumab vs. Placebo) in the multivariable logistic regression analysis was adjusted for Age (per year), Sex (Male vs. Female), Body mass index (per kg/m^2^), Smoking (Former or current vs. Never), Family history of psoriasis (Yes vs. No), Previous local treatment (Yes vs. No), Previous non-biological agent treatment (Yes vs. No), Previous biological agent treatment (Yes vs. No), Previous phototherapy (Yes vs. No), Hypertension (Yes vs. No), Diabetes mellitus (Yes vs. No), Duration of psoriasis (per year), Psoriasis area and severity index score (per score), Static physician's global assessment score (4 vs. 3; 5 vs. 3), body surface area (per %), Dermatology life quality index score (per score).

#, Reduced covariate set: Randomized treatment assignment (Vunakizumab vs. Placebo) in the multivariable logistic regression analysis was adjusted for Age (per year), Sex (Male vs. Female), Duration of psoriasis (per year), Static physician's global assessment score (4 vs. 3; 5 vs. 3).

※, Model 1, skin and subcutaneous tissue disorders containing psoriasis exacerbation; Model 2, skin and subcutaneous tissue disorders after excluding psoriasis exacerbation.
